# Role Overload, Perceived Gratitude, and Health Among Spousal Caregivers: A Longitudinal Study

**DOI:** 10.1093/geroni/igaa057.1137

**Published:** 2020-12-16

**Authors:** Suyoung Nah, Lynn Martire

**Affiliations:** 1 The Pennsylvania State University, State College, Pennsylvania, United States; 2 The Pennsylvania State University, University Park, Pennsylvania, United States

## Abstract

Spouses are likely the most vulnerable caregivers due to their advanced age and more intense caregiving than other family members. Thus, it is crucial to identify risk and protective factors for spousal caregivers’ health. According to Pearlin’s stress process model, caregivers’ subjective stressors and resources may impact their health. We focused on spousal caregivers’ role overload, a critical indicator of subjective stressors, and their perception that the partner is grateful for their help, which may be beneficial for their health but has rarely received attention. This study tested the hypotheses that spousal caregivers’ higher role overload and lower perceived gratitude are associated with poorer physical and mental health over time. We also examined whether greater perceived gratitude buffers the negative relationships between role overload and health. We focused on 223 spousal caregivers of older adults without dementia from the 2015 and 2017 National Study of Caregiving. Autoregressive models revealed that spousal caregivers’ higher role overload at baseline was associated with poorer self-rated health at follow-up (b = -0.23, p < .05), but not with depressive symptoms or anxiety. Spousal caregivers’ greater perceived gratitude at baseline was associated with lower anxiety at follow-up (b = -0.32, p < .01). There were no moderating effects of perceived gratitude on the relationships between role overload and health. These findings suggest that spousal caregivers’ role overload is a risk factor for their physical health, while their perception that the partner is grateful for their help serves as a protective factor for their mental health.

